# Proximal Tibiofibular Joint Surgery: Preoperative Planning With MRI Scan to Determine Intraoperative Safe Zones

**DOI:** 10.7759/cureus.89978

**Published:** 2025-08-13

**Authors:** Amirzeb Aurangzeb, Wen Loong Paul Yuen, Koh Xuan Han, Raghuraman Raghavan, James Loh Sir Young

**Affiliations:** 1 Department of Orthopaedic Surgery, Changi General Hospital, Singapore, SGP; 2 Department of Health Sciences Research, Changi General Hospital, Singapore, SGP

**Keywords:** fibula, knee, posterolateral corner reconstruction, proximal tibiofibular joint, tibia

## Abstract

Background: The proximal tibiofibular joint (PTFJ) is a less-understood aspect of the knee, which has significance in knee injuries. However, current literature has shown that there is a lack of a gold standard in PTFJ fixation. Our study aims to characterize the PTFJ and describe safe zones for intraoperative instrumentation and implant fixation for PTFJ stabilization.

Methods: Magnetic resonance imaging (MRI) knee images from 49 patients in a large public institution were reviewed retrospectively and analysed. Morphological parameters of PTFJ were measured, such as the PTFJ angle, width, shape, and safe zones for instrumentation. Descriptive statistics of parameters were reported as mean ± standard deviation (SD), with 95% confidence intervals (CI), and the Mann-Whitney U-test was used to examine if there were differences in PTFJ parameters between male and female patients.

Results: The PTFJ angle had a mean of 28.2 ± 6.5° on the coronal view, 29.5 ± 6.6° on the sagittal view, and 34.2 ± 6.8° on the axial view. PTFJ width had a mean of 15.4 ± 1.8mm on the coronal view, 18.3 ± 2.2mm on the sagittal view, and 17.0 ± 1.7mm on the axial view. Male patients were found to have a wider PTFJ width on the coronal (P = 0.015), sagittal (P = 0.002), and axial (P = 0.008) planes when compared to female patients. The superior, anterior, and posterior safe zones for PTFJ instrumentation were 14.3° ± 2.5, 36.3° ± 6.1, and 6.8° ± 6.6, respectively. Male patients were found to have a longer distance from the fibula head to the entry point on both the coronal (P = 0.001) and sagittal (P = 0.004) planes. The mean distance from the neurovascular bundle was 12.6 ± 1.9mm.

Conclusions: In our study, we defined safe zones for PTFJ stabilization and described our intraoperative technique for instrumentation and implant fixation of the PTFJ joint to minimize complications. We also described morphological parameters of the PTFJ for our study population. The study will hopefully contribute to further understanding of the PTFJ and describe intraoperative safe zones for the stabilization of PTFJ injuries.

## Introduction

The proximal tibiofibular joint (PTFJ) constitutes a synovial joint formed by the articulation between the fibular facet on the superomedial surface of the fibula head and the tibial facet located on the posterolateral aspect of the tibial condyle [1]. Despite being a less understood aspect of the knee, recent research has shed light on its significance, prompting a growing acknowledgment of the PTFJ as a potential fourth compartment within the knee joint [1]. PTFJ anatomy has also been thought to play a role in the development of knee osteoarthritis [1-3].

PTFJ injuries account for less than 1% of all knee injuries and 9% of multi-ligamentous knee injuries [4]. The mechanism of injury typically entails torsion of the knee with supination of the foot, which can be the result of a fall, sporting injury, or direct high-energy trauma [5]. Unrecognized and untreated PTFJ injuries can be associated with acute and chronic lateral-sided knee pain, fibular head subluxation or dislocation, and even failure of posterolateral corner (PLC) reconstruction [4]. Anatomical structures that form the PLC include the fibular collateral ligament, biceps femoris, popliteal-fibular ligament, and arcuate ligament, which insert onto the fibular head. Hence, a stable PTFJ is required for successful PLC reconstruction, especially for fibula-based techniques [4,6].

Various surgical techniques have been described for PTFJ stabilization, and these include screw fixation, graft reconstruction, and suspensory device fixation [6,7]. The technical challenges include limited working space and risk of collision between the grafts, implants, and bony tunnels [6]. A good working knowledge of the anatomy and safe zones of the PTFJ during surgery is thus critical in optimizing a surgical technique. This retrospective MRI-based study aims to define safe zones for instrumentation and fixation of the PTFJ to guide surgical stabilization. By analyzing high-resolution knee MRI scans from a defined patient cohort, the study seeks to guide preoperative planning and minimize intraoperative complications, particularly neurovascular injury

## Materials and methods

This study was conducted in the orthopaedic department of Changi General Hospital, Singapore. The study was approved by Singhealth Centralised Institutional Review Board (approval number: 2021/2257), which included the waiver of informed consent. All methods were performed in accordance with relevant guidelines and regulations.

Study subjects

A total of 49 patients underwent simple random sampling from a pre-existing database of orthopaedic patients with knee MRI performed. A computer-generated random number sequence was applied to the anonymised list of patients to ensure unbiased selection. A preliminary pilot study with statistical consultation was performed on a smaller subset of patients. The lowest intraclass correlation coefficient (ICC) values in the pilot ranged between 0.55 and 0.68, depending on the parameters and MRI planes. Following standard guidelines for ICC reliability studies, we targeted a minimum ICC threshold of 0.5 (moderate reliability) to 0.7 (good reliability) as the acceptable range with a significance level of 0.05 and 80% power, with a minimum of 30 knees recommended. Three independent surgeons, each blinded to patient information and to the datasets of the other surgeons, retrospectively reviewed and analyzed the MRI images. Clinical indications for MRI in these patients include knee pain, minor trauma, or sports-related injuries. Exclusion criteria included age less than 16 years, history of inflammatory arthropathy, cancer, previous knee surgery, or high-energy knee trauma. 

MRI Imaging

Knee MRI scans were performed using 1.5-T whole body magnetic resonance unit (Siemens Aera and Siemens Sola; Siemens AG, Munich, Germany) using sagittal, axial and coronal sequences with slice thickness 3.0 mm with no gap, repetition time >3500 ms, echo time <50 ms; 512x512 pixel matrix, sagittal T2 Dixon sequence with slice thickness 3.0 mm with no gap, repetition time >2500 ms, echo time 55-70 ms.

Morphological parameters of PTFJ measurements

The PTFJ morphological measurements were taken with the above-mentioned MRI sequences. Using the MRI coronal cuts, parameters of PTFJ angle, PTFJ width, safe zone for superior angle of instrumentation, and distance from tip of fibula head to instrumentation entry point were measured. Using the MRI sagittal cuts, parameters of PTFJ angle, PTFJ width, distance from the tip of the fibula head to the instrumentation entry point, and configuration of PTFJ shape were measured. Using the MRI axial cuts, parameters of PTFJ angle, PTFJ width, safe zone for anterior and posterior angles of instrumentation, and distance from the posterior knee neurovascular bundle were measured.

MRI protocol

Coronal View

The corresponding coronal and axial views were used to determine the lateral-most and clinically suitable entry point for a guide wire. From that point, a horizontal line was drawn on the coronal view. A 10 mm mark distal to the bone-articular cartilage junction was indicated on the far tibia cortex. A second line was drawn from the entry point on the fibular head to the mark on the medial tibia cortex. The angle between the two lines was then determined. The coronal safe zone was the area between these two lines. The width and gradient of the PTFJ articular surface, as well as the distance from the fibular tip, were measured in the same coronal view.

Sagittal View

The sagittal view with the largest PTFJ articular surface was chosen. The level of the entry point for the guide wire on the previously used coronal cut was translated onto this sagittal view. The distance from the tip of the fibular head to the wire entry point was measured. The width and gradient of the articular surface on this sagittal view were measured.

Axial View

The axial view that corresponded with the coronal view used for measuring the coronal safe zone was retained for use. Two lines were used to mark out the axial safe zone. The anterior line was projected from the lateral entry point to the medial border of the tibial tuberosity. Similarly, the posterior line was projected to touch the medial-most point on the tibial medial cortex. The area between these two lines is the axial safe zone. The width of the PTFJ articular surface and the perpendicular distance of the neurovascular bundle from the posterior line of the axial safe zone were measured.

**Figure 1:**
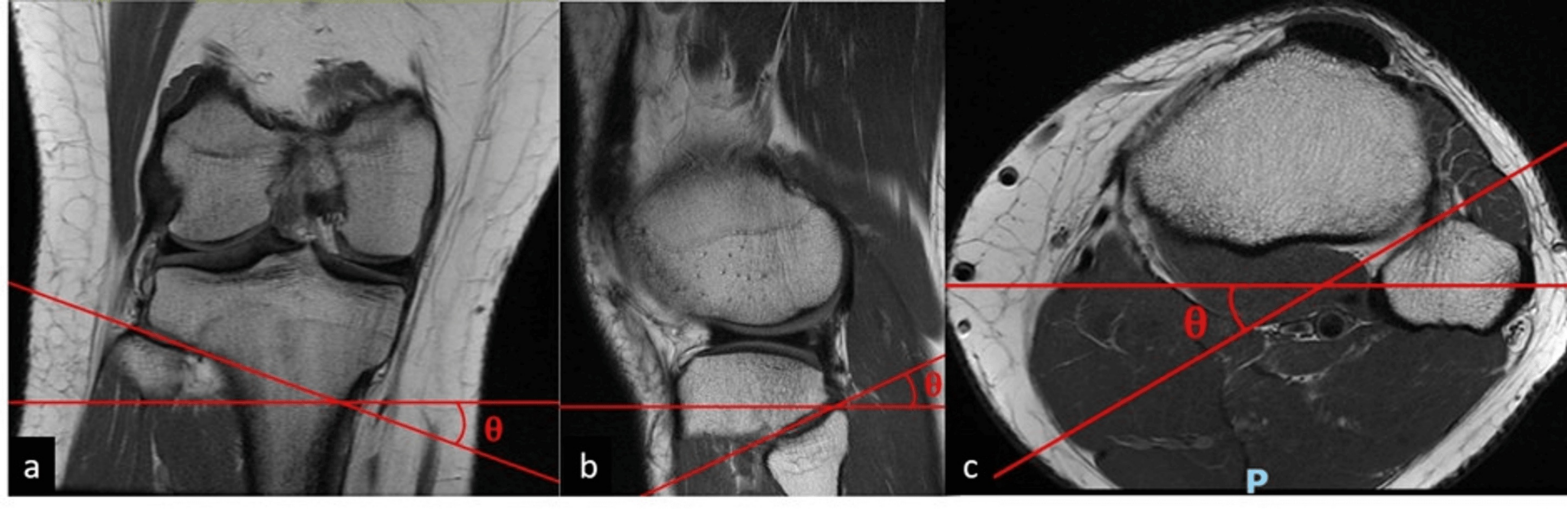
MRI imaging of the right knee in the coronal (a), saggital (b), and axial (c) views. The proximal tibiofibular joint angle (θ), demarcated in red lines, is measured against the horizontal axis.

**Figure 2:**
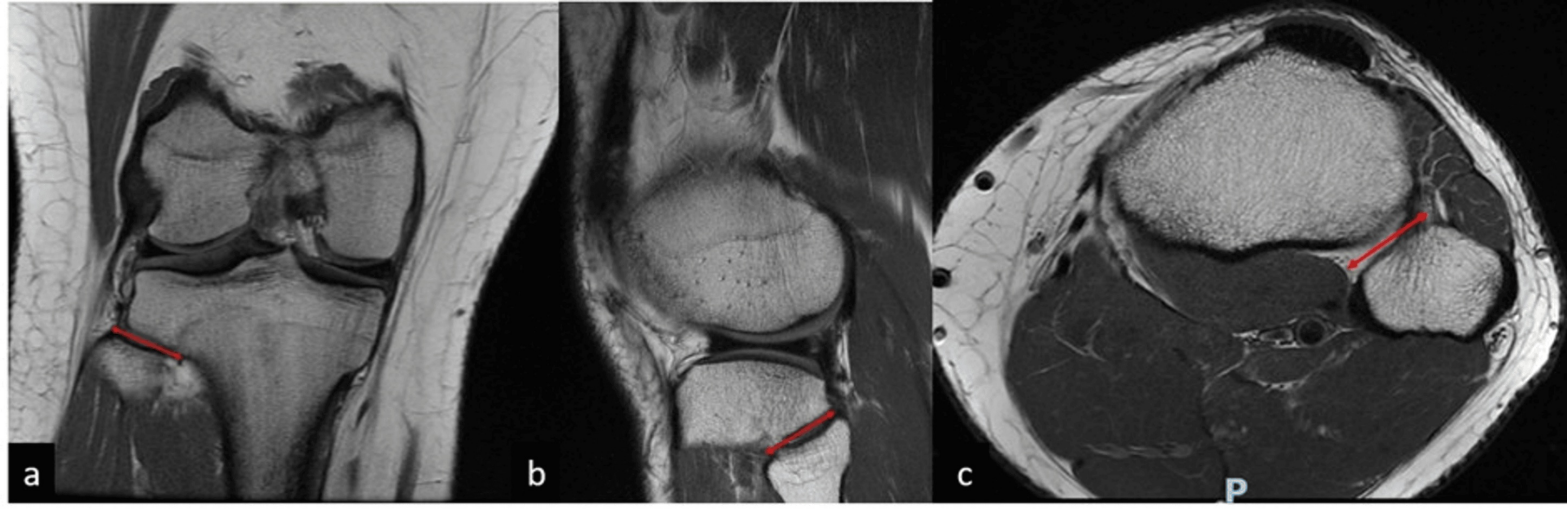
MRI imaging of the right knee in the coronal (a), saggital (b), and axial (c) views. The proximal tibiofibular joint width, demarcated in arrowed red lines is measured.

**Figure 3:**
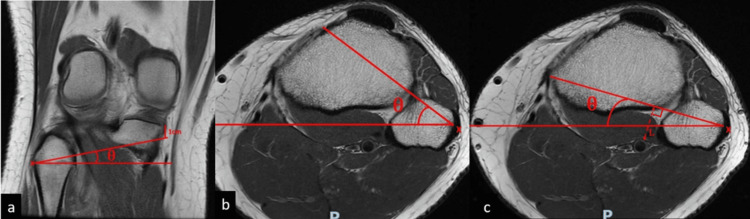
MRI imaging of the right knee in the coronal (a), and axial (b,c) views. The angles of superior, anterior, and posterior safe zones for PTFJ (θ), demarcated in red lines are measured against the horizontal. The superior safe zone for instrumentation was measured on the coronal cuts (a), to a pre-identified point 1 cm below the medial most aspect of the joint line. The anterior safe zone is measured on the axial cuts (b), to the medial most point of the tibial tubercle. The posterior safe zone is measured on the axial cuts (c), to the medial most point on the medial proximal tibia cortex. The nearest distance (L) between the posterior safe zone and the neurovascular bundle is measured.

**Figure 4:**
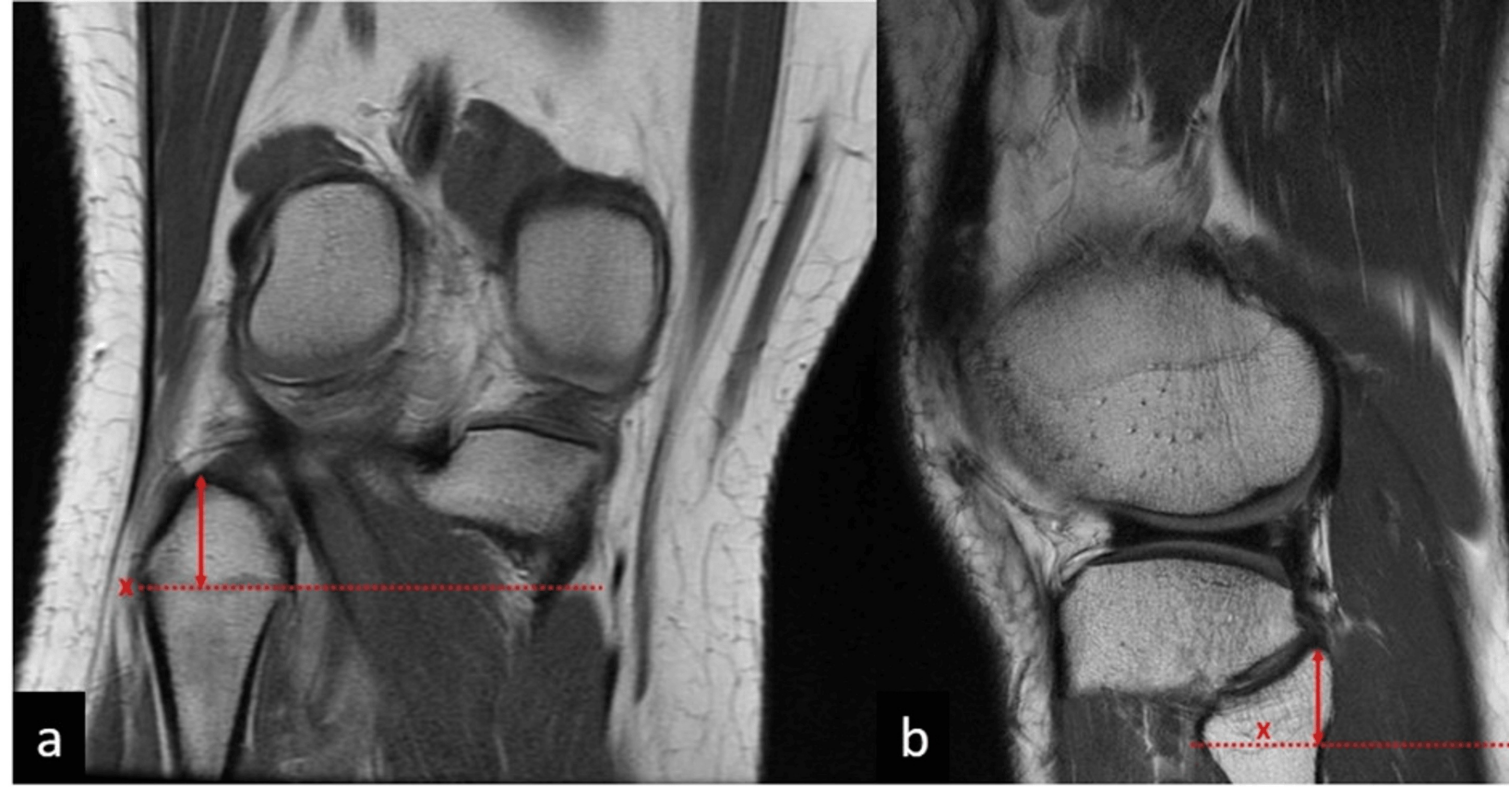
MRI imaging of the right knee in the coronal (a) and saggital (b) views. The distance between the tip of the fibula head and the pre-determined entry point (marked x), demarcated in arrowed red lines is measured.

In summary, the PTFJ angle was measured on the coronal, axial, and sagittal views against the horizontal axis (Figure 1). The width of the PTFJ was measured on the three orthogonal views (Figure 2). The superior and axial safe zones for PTFJ were identified (Figure 3). The distance between the tip of the fibula head and the pre-determined entry point is demarcated (Figure 4). The ideal entry point for PTFJ instrumentation (specifically for screw or suspensory device fixation) was identified as the most lateral point of the fibula head. All angular measurements on the three orthogonal planes were corrected for tibial tilt by first identifying the tibial shaft axis, drawing a line through the midpoint of two transverse lines through the tibial diaphysis. All angular measurements were then taken relative to a horizontal line perpendicular to this tibial axis. This is important as the tibia itself is taken as the reference point during intraoperative instrumentation, and hence any angular measurements should be referenced off the tibia axis instead of the longitudinal or horizontal axis of the MRI imaging.

Statistical analysis

The data collection was performed by three independent orthopaedic surgeons using Vue Motion Viewer (Carestream Health, Rochester, New York, United States). ICC was calculated to assess for interobserver reliability. Of all measured PTFJ parameters, the majority (85.7%) achieved moderate to excellent reliability. Descriptive statistics of all PTFJ parameters were reported as mean ± standard deviation (SD), with corresponding 95% confidence intervals (CIs) for the mean. The Mann-Whitney U-test was used to examine if there were differences in PTFJ parameters between male and female patients. Statistical tests were two-sided with a 0.05 significance level. All statistical analyses were conducted using Stata Statistical Software: Release 15 (StataCorp LLC, College Station, Texas, United States).

## Results

A total of 52 knees (20 left, 32 right) of 49 patients were included in the analysis. The mean age of the patients was 25.6±6.7 and 89.8% (44/49) were male.

Morphological PTFJ parameters

Across the coronal, sagittal, and axial planes, mean PTFJ angles were relatively similar, and no statistically significant gender differences in angulation were observed, as seen in Table 1. In contrast, PTFJ width was consistently greater in male patients across all planes, indicating a possibly wider bony corridor for instrumentation in this group. Interobserver reliability for PTFJ angle measurement ranged from ICC 0.55 (coronal) to 0.80 (sagittal) and 0.70 (axial), reflecting moderate to good agreement. Width measurements showed moderate reliability in the coronal (ICC 0.50) and sagittal planes (ICC 0.42), but lower agreement in the axial plane (ICC 0.23), likely reflecting challenges in precisely delineating joint margins in that view.

**Table 1:** MRI morphological parameters of the PTFJ in orthogonal views of coronal, sagittal and axial planes PTFJ: proximal tibiofibular joint

Morphological PTFJ Parameters	Coronal		Sagittal		Axial	
	Mean ± SD	95% CI of mean	Mean ± SD	95% CI of mean	Mean ± SD	95% CI of mean
PTFJ angle (deg.)						
All knees	28.2 ± 6.5	26.4 to 30.1	29.5 ± 6.6	27.6 to 31.3	34.2 ± 6.8	32.3 to 36.1
Male	28.5 ± 6.7	26.5 to 30.4	29.4 ± 6.5	27.5 to 30.3	33.9 ± 8.0	31.6 to 36.3
Female	26.0 ± 4.3	20.6 to 31.4	29.8 ± 7.7	20.2 to 39.4	33.2 ± 5.3	26.6 to 39.8
PTFJ width (mm)						
All knees	15.4 ± 1.8	14.9 to 15.9	18.3 ± 2.2	17.7 to 19.0	17.0 ± 1.7	16.6 to 17.5
Male	15.5 ± 1.8 (p=0.015)	15.0 to 16.1	18.6 ± 2.1 (p=0.002)	18.0 to 19.3	17.2 ± 1.7 (p=0.008)	16.7 to 17.7
Female	13.6 ± 1.0	12.4 to 14.8	15.6 ± 0.7	14.7 to 16.6	15.3 ± 0.8	14.4 to 16.3

Morphological PTFJ shape

The most common PTFJ morphologies in this cohort were the plane and trochoid types, together comprising nearly two-thirds of all cases, as seen in Table 2. The remaining third consisted of irregular configurations such as double trochoid, condylar, saddle, trochlear, and ball-and-socket types. The distribution was similar between male and female patients, although the small number of female patients limited definitive comparison. 

**Table 2:** Distribution of morphological PTFJ shapes PTFJ: proximal tibiofibular joint

Morphological PTFJ Shape	Plane, n (%)	Trochoid, n (%)	Double Trochoid, n (%)	Condylar, n (%)	Saddle, n (%)	Trochlear, n (%)	Ball and Socket, n (%)
All knees	17 (32.7%)	17 (32.7%)	10 (19.2%)	4 (7.7%)	1 (1.9%)	1 (1.9%)	2 (3.8%)
Male	15 (31.9%)	16 (34.0%)	8 (17.0%)	4 (8.5%)	1 (2.1%)	1 (2.1%)	2 (4.2%)
Female	2 (40.0%)	1 (20.0%)	2 (40.0%)	-	-	-	-

Parameters for PTFJ instrumentation

All safe zone angles in our study were measured relative to the tibial axis rather than the default horizontal reference in the MRI image. This was achieved by correcting for tibial tilt in each plane, using the tibial shaft as the anatomical reference line. This method ensures that the reported angles directly correspond to the orientation surgeons encounter intraoperatively when aligning instruments to the tibia. Safe zone measurements for superior, anterior, and posterior trajectories showed no significant gender differences, indicating that these angular corridors are generally consistent across patients, as seen in Table 3. However, the distance from the fibular head tip to the instrumentation entry point was longer in male patients, reflecting their larger bony dimensions. The mean separation between the posterior margin of the axial safe zone and the neurovascular bundle was consistent across genders, providing a predictable safety buffer during instrumentation. Reliability for safe zone measurements was moderate: ICC 0.63 for the superior angle, ICC 0.61 for the anterior safe zone angle, and ICC 0.83 for the posterior safe zone angle. The distance from the fibular head tip to the entry point had ICCs of 0.65 (coronal) and 0.57 (sagittal), while the neurovascular bundle distance achieved good reliability (ICC 0.71). These findings suggest that while safe zone orientation is largely similar between sexes, variations in bony size should be considered during preoperative planning, particularly when selecting implant size and trajectory.

**Table 3:** MRI parameters for PTFJ instrumentation PTFJ: proximal tibiofibular joint

Parameters for PTFJ instrumentation	Mean ± SD	95% CI of mean
Safe zone for superior angle (deg.)		
All knees	14.3 ± 2.5	13.6 to 15.1
Male	14.2 ± 2.5	13.5 to 14.9
Female	15.7 ± 2.3	12.9 to 18.6
Safe zone for anterior angle (deg.)		
All knees	36.3 ± 6.1	34.6 to 38.0
Male	36.2 ± 6.3	34.3 to 38.0
Female	37.5 ± 5.2	31.1 to 43.9
Safe zone for posterior angle (deg.)		
All knees	6.8 ± 6.6	5.0 to 8.7
Male	6.5 ± 6.5	4.6 to 8.4
Female	9.9 ± 7.4	0.6 to 19.1
Distance from neurovascular bundle (mm)		
All knees	12.6 ± 1.9	12.1 to 13.1
Male	12.6 ± 2.0	12.0 to 13.2
Female	12.5 ± 1.8	10.3 to 14.7
Distance from tip of fibula head to entry point, coronal (mm)		
All knees	16.4 ± 2.4	15.8 to 17.1
Male	16.8 ± 2.3 (p=0.001)	16.1 to 17.4
Female	13.1 ± 0.7	12.3 to 14.0
Distance from tip of fibula head to entry point, sagittal (mm)		
All knees	16.7 ± 2.0	16.2 to 17.3
Male	17.0 ± 1.9 (p=0.004)	16.4 to 17.5
Female	14.3 ± 1.4	12.6 to 16.1

## Discussion

According to current literature, surgical treatment of PTFJ instability leads to restored function, symptom relief, and improved subjective knee scores [7]. However, there is a diverse array of surgical options due to the lack of a gold standard, emphasizing the need to expand the current understanding of the PTFJ. The current study determined the entry point on the fibular head and safe zones to guide PTFJ instrumentation and implant fixation of the PTFJ. These parameters are applicable to screw fixation, suspensory devices, and surgery in this region. The distance between the entry point of the guide wire on the most lateral part of the fibular head surface and the palpable fibular tip is 16.7 mm ± 2 (SD) on the sagittal plane and 16.4 mm ± 2.4 (SD) on the coronal plane. This facilitates the placement of the guide wire in the thicker part of the fibular head after exposure. The coronal zone has a mean of 14.3° +/- 2.5° (SD) from the horizontal to 10mm from the articular surface in the same plane. When the angulation in the corresponding axial view and a positive tibia slope are factored into consideration, this measured safe zone is generally less than the intraoperative safe zone and further reduces the risk of injury to the medial tibia plateau articular surface and meniscus. The axial safe zone has a mean of 21.55° ± 6.35 (SD) (an overlap of the anterior safe zone of 36.3° ± 6.2, and posterior safe zone of 6.8° ± 6.6 from the horizontal) and the posterior neurovascular bundle at a mean distance of 12.6 mm ± 1.9 (SD) from the posterior margin of the axial safe zone. Working within this zone reduces the chance of injury to the tibial tuberosity, patella tendon, and neurovascular bundle.

There are reported variations in morphological features such as joint inclination angle, articular surface area, and shape, and their implications [8]. Ogden classified PTFJ into oblique and horizontal types (less than 20-degree inclination) [9]. PTFJ with horizontal morphology were associated with more rotational mobility and less prone to injury, while oblique-oriented PTFJ have a smaller articular surface area and reduced ability to tolerate torsional forces [8,9]. Suga et al. found that horizontal type PTFJ was significantly associated with discoid lateral meniscus [10]. On the other hand, Eichenblat and Nathan further characterized PTFJ morphology into seven different subtypes based on their cadaveric study; plane (33.55%), trochoid (29.57%), double trochoid (22.59%), condylar (4.65%), saddle (2.32%), trochlear (.86%) and ball and socket (.67%) [11].

Lu et al. observed that patients with irregular-shaped PTFJ (non-plane or trochoid) were more likely to exhibit osteoarthritic changes in lateral tibial and femoral sites [1], and, as Zhao et al.'s study finds, increases the risk of subsequent total knee arthroplasty [12]. The morphological variations in the current study are similar. The mean PTFJ inclination of 28.2°, 29.5°, and 34.2° in the coronal, sagittal, and axial planes, respectively, is similar to that in the study by Huang et al., in which they found that a lower PTFJ inclination was associated with risk of medial compartmental knee osteoarthritis [2]. In their study, the inclination of the PTFJ was measured for the tibia and fibula separately on the lateral plain radiographs and was 33.44 and 31.99, respectively, for the control group (without pre-existing knee osteoarthritis), and 26.91 and 26.82, respectively, for the patient group (with knee osteoarthritis). The similarities in the PTFJ morphology of the current study with previous reports suggest favorably the potential application of the proposed measurement method.

Factoring in the different joint surface morphology previously described and the safe zones outlined, it is extrapolated that the guide wire and fixation device tract is not likely to penetrate the articular surface. Though the above-described parameters serve as a guide during surgery, it is important to keep in mind the anatomical variation of each patient. The described measurement method needs to be applied accordingly during preoperative planning to determine the parameters specific to each patient. It needs to be borne in mind that the MRI imaging, offering a static viewpoint with the knee in full extension, does not fully capture the dynamic changes in the posterior neurovascular bundle position, which recedes further posterolateral with knee flexion during surgery.

A significant gender difference, with the PTFJ width and distance from the tip of the fibula head to the entry point being larger in male subjects, is attributed to the wider fibula head diameter and corresponding tibia facet. The current study found that the average width of PTFJ was 15.4, 18.3, and 17.0mm on the coronal, sagittal, and axial planes, respectively, indicating the inherently limited working space.

There are several limitations to our retrospective study. We note a significant male predominance in our study. Though strict screening was performed to exclude pathology that would affect the result, it involved a heterogeneous patient population seeking treatment for diverse knee conditions, ranging from knee pain to minor trauma and sports-related injuries. In future studies, it would be preferable to select MRI scans from a cohort of healthy young volunteers devoid of pre-existing knee symptoms to minimize potential confounding factors. The medial-most point on the tibia medial cortex, used in delineating the posterior safe zone, is arbitrarily chosen as it is the most posterior point on the medial surface. Instrumentation posterior to this point is likely challenging due to the obliquity of the posterior tibia cortex in sagittal and axial planes. Another constraint in our analysis pertains to the quality of the MRI imaging, specifically, slice thickness and image resolution. The suboptimal ICC values for morphological parameters of axial and sagittal PTFJ width may be attributed to challenges in precisely delineating the joint margin on the MRI scans. A consideration would be for addition of a specific MRI PTFJ series with cuts taken in line with the joint for better visualization. Addressing these limitations in future studies through more balanced participant demographics and enhanced imaging quality would undoubtedly strengthen the robustness and applicability of the findings. Lastly, subsequent studies should also consider the use of dynamic imaging to overcome the limitations posed by the static imaging used in our study. 

## Conclusions

This anatomical study presents a practical preliminary method for preoperative planning of PTFJ surgery using MRI imaging. By quantifying anatomical parameters such as joint angle, width, and safe instrumentation zones across coronal, sagittal, and axial planes, we provide a detailed framework for safer and more effective surgical intervention. The study also highlights gender-based differences in morphometric measurements and establishes normative data within a Southeast Asian population, which remains underrepresented in current literature. The proposed MRI-based preoperative planning approach enables individualized instrumentation trajectories, potentially improving surgical accuracy and reducing complications. Future research should aim to validate these safe zones through cadaveric or intraoperative studies, assess their impact on clinical outcomes, and explore the development of imaging-guided planning tools. The described technique may also serve as a foundation for further exploration into PTFJ biomechanics and variations in morphology across populations. 
